# ESCRT Requirements for Murine Leukemia Virus Release

**DOI:** 10.3390/v8040103

**Published:** 2016-04-18

**Authors:** Christina Bartusch, Reinhild Prange

**Affiliations:** Department of Medical Microbiology and Hygiene, University Medical Center of the Johannes Gutenberg University Mainz, Augustusplatz, Mainz D-55131, Germany; bartusc@students.uni-mainz.de

**Keywords:** MLV, VLPs, retroviral budding, viral late domain, ESCRT, MVB pathway, CHMP1A

## Abstract

The Murine Leukemia Virus (MLV) is a gammaretrovirus that hijack host components of the endosomal sorting complex required for transport (ESCRT) for budding. To determine the minimal requirements for ESCRT factors in MLV viral and viral-like particles (VLP) release, an siRNA knockdown screen of ESCRT(-associated) proteins was performed in MLV-producing human cells. We found that MLV VLPs and virions primarily engage the ESCRT-I factor Tsg101 and marginally the ESCRT-associated adaptors Nedd4-1 and Alix to enter the ESCRT pathway. Conversely, the inactivation of ESCRT-II had no impact on VLP and virion egress. By analyzing the effects of individual ESCRT-III knockdowns, VLP and virion release was profoundly inhibited in CHMP2A- and CHMP4B-knockdown cells. In contrast, neither the CHMP2B and CHMP4A isoforms nor CHMP3, CHMP5, and CHMP6 were found to be essential. In case of CHMP1, we unexpectedly observed that the CHMP1A isoform was specifically required for virus budding, but dispensable for VLP release. Hence, MLV utilizes only a subset of ESCRT factors, and viral and viral-like particles differ in ESCRT-III factor requirements.

## 1. Introduction

An essential step in the formation of an extracellular enveloped virus particle is the separation of virus and host cell membranes. For many viruses, this requires the recruitment of a network of proteins normally involved in analogous cellular membrane fission events, like intraluminal vesicle formation at the multivesicular body (MVB), separation of daughter cells during cytokinesis, and extracellular shedding of plasma membrane wounded portions [1,2,3,4,5,6]. This network, collectively called ESCRT (endosomal sorting complex required for transport), consists of heteromeric ESCRT-0, -I, -II, and -III complexes together with the VPS4 ATPase that seem to function in a sequential manner [1,4,7]. The upstream factors ESCRT-0 and ESCRT-I recognize and concentrate cargoes, whereas the later-acting factors ESCRT-III mediate membrane constriction and scission events and recruit the terminal VPS4 ATPase complex for disassembly and recycling of the ESCRT machinery. ESCRT-II is supposed to connect the upstream cargo-binding components of the system with the downstream membrane remodeling system [8,9].

As ESCRT-mediated fission events resemble enveloped virus budding, a wide variety of viruses use ESCRT pathway functions to escape from the cell. Viruses recruit ESCRT components through the function of specific peptide motifs within their structural proteins referred to as late assembly domains [10,11]. The first late domain to be identified and characterized was the P(S/T)AP motif present in the p6 region of the Gag protein of human immunodeficiency virus type 1 (HIV-1) [12]. The disruption of this region resulted in defective virus budding, characterized by vesicles that remained tethered to the plasma membrane. For the Rous sarcoma virus (RSV) Gag protein, a PPXY motif was identified to contribute late in the budding process, while the Ebola virus takes use of overlapping P(S/T)AP and PPXY domains [10,11,13]. Similar tetra-amino acid sequences characterized subsequently include the (L)YPX_n_L and FPIV motifs found in different viruses [10,11]. These sequences are collectively termed late domains to reflect their function late in the virus budding process. All viruses that bud in an ESCRT-dependent manner share the requirement for the membrane abscission function provided by ESCRT-III and VPS4, but differ in their need for upstream-acting factors. Viruses relying on P(S/T)AP late motifs, such HIV-1, Ebola, and human T-cell leukemia virus type 1, engage the pathway via interaction with the ESCRT-I component Tsg101 [3,5,7,14]. In analogy to MVB biogenesis, ESCRT-I may then recruit ESCRT-II that binds the ESCRT-III subunit CHMP6 thereby leading to recruitment and assembly of the ESCRT-III/VPS4 scission machinery [1,9]. The need of ESCRT-II in P(S/T)AP-dependent budding is still a matter of debate, as there are conflicting data whether HIV-1 egress requires this complex or not [8,15,16]. Assuming that ESCRT-II and CHMP6 are dispensable for HIV-1 budding, it remains unclear how the virus triggers ESCRT-III assembly via ESCRT-I. Conversely, the human hepatitis B virus (HBV) and the avian sarcoma virus (ASV) do not depend on ESCRT-I, but rather utilize ESCRT-II to enter and activate the ESCRT-III/VPS4 complex [17,18]. The (L)YPX_n_L-type late domains found in equine infectious anemia virus (EIAV) and Sendai virus are responsible for binding Alix, an ESCRT-associated protein that links viral proteins to the CHMP4 subset of the downstream ESCRT-III complex, thereby bypassing the need of ESCRT-I and ESCRT-II [3,5]. PPXY-type late domains present in viruses such as RSV, murine leukemia virus (MLV), and Ebola virus access the ESCRT machinery via interaction with members of the Nedd4-like HECT ubiquitin ligase family. Connections between PPXY-dependent budding and ESCRT-III/VPS4 recruitment are less firmly established, but appear to involve ubiquitin, the ubiquitin ligase activity of the HECT proteins, and ubiquitin-binding components [10,19,20,21,22]. The identity of the relevant ubiquitin acceptor(s) has not been clearly established, but may comprise ubiquitin molecules attached to viral proteins that could recruit ubiquitin-binding factors of the ESCRT pathway, such as Tsg101, the ESCRT-II subunit EAP45, or Alix [1,4]. Alternatively, self-ubiquitination of HECTs could provide a platform for ubiquitin-dependent ESCRT recruitment. In addition, arrestin-related trafficking (ART) proteins could provide a physical link between HECTs and ESCRT, as they are substrates for HECT-mediated ubiquitination and inhibit PPXY-dependent virus budding, if overexpressed [21]. Auxiliary interactions in viral budding have been described for Nedd4-type ubiquitin ligase isoforms (Nedd4-2s) and HIV-1 Gag, although HIV-1 lacks PPXY motifs. In this recruitment pathway, the *N*-terminal C2 domain, rather than the PPXY-binding WW domain of Nedd4-2s functions as an autonomous Gag-targeting module, thereby stimulating HIV-1 budding [23,24].

Many viruses contain multiple late domains that can bind and recruit different early-acting ESCRT or ESCRT-associated factors. Due to this redundancy, the according entry sites into ESCRT pathway axes remain to be dissected. For example, murine leukemia viruses, prototypical gammaretroviruses, encode all three canonical late motifs in their Gag polyproteins with the PPPY domain being essential, while the PSAP and LYPAL motifs play complimentary roles [19,25,26]. As with other retroviruses, ESCRT-dependent budding of MLV is driven by the Gag polyprotein that alone is able to induce the formation and release of membrane-coated, virus-like particles (VLPs) even in the absence of viral envelope proteins [26,27,28]. The functional requirements for intermediate- and late-acting ESCRT factors in MLV budding have not been dissected in detail. We therefore investigated the impact of different ESCRT factors and their isoforms on the egress of MLV viral and viral-like particles. Given that MLV is an important model system to study the usefulness of retrovirus vectors in gene-transfer trials [29], the elucidation of the budding pathways of its particle types may also be instrumental.

## 2. Materials and Methods

### 2.1. Expression Constructs

The expression vector encoding the MLV Gag full-length precursor protein with a *C*-terminal fused, enhanced yellow fluorescent protein (YFP) (termed herein MLV.Gag^YFP^) was generously provided by Mothes, *et al.* [28] via Addgene (plasmid #1813). The retroviral packaging vector pCL-Eco containing the MVL Gag/Polymerase (Pol) and Envelope (Env) open reading frames (ORFs) under the control of the CMV immediate/early promoter was a gift from Inder Verma [30] and obtained from Addgene (plasmid #12371). For tagging of the Gag protein with a FLAG epitope, we co-opted a strategy described by Yueh and Goff [31], whereupon the amino acid (aa) sequence GNGGEAT of the p12 domain (aa 177 to 183, numbering as referred to the Moloney murine leukemia virus genome, Accession number NC_001501) was substituted by YKDDDDK. For mutagenesis, the Q5^®^ Site-Directed Mutagenesis Kit (New England Biolabs, Frankfurt/Main, Germany) was used. The primers Gag.FLAG.forward (5′-TCCGACAGGGAC**TAT**AA**G**G**A**TG**AT**GA**C**G**AT**A**AG**C**T**TGCGGGAGAGGCACCG-3′; mutant nucleotides are shown in boldface) and Gag.FLAG.reverse (5′-CGGTGCCTCTCCCGCA**A**G**CT**T**AT**C**G**TC**AT**CA**T**C**C**TT**ATA**GTCCCTGTCGGA-3′) were designed using the NEBaseChanger™ software (v1.2.4, New England Biolabs). Mutagenesis was performed in the MLV.Gag background (termed herein MLV.Gag^FLAG/YFP^) and in the MLV retroviral construct. In addition, the Env ORF encoded by pCL-Eco was tagged with a HA-epitope (YPYDVPDYA) that was inserted between aa 294 and 295 of Env. The insertion site is located within a proline-rich region connecting the *N*-terminal receptor binding domain and the *C*-terminal transmembrane domain of Env and had been shown to be permissive for insertions [32]. For mutagenesis, the primers Env.HA.forward (5′-CCTTCAGTCACCAAACCA**TACCCATACGATGTTCCAGATTACGCT**CCCAGTGGGACT**G**C**AG**TCTCCCCT-3′; mutant nucleotides are shown in boldface) and Env.HA.reverse (5′-AGGGGAGA**CT**G**C**AGTCCCACTGGG**AGCGTAATCTGGAACATCGTATGGGTA**TGGTTTGGTGACTGAAGG-3′) were used. The modified pCL-Eco construct is termed MLV.WT^FLAG/HA^ herein. The pCL-MFG-LacZ reporter construct used for packaging in the tagged MLV retroviral pCL-Eco vector was purchased from Novus Biologicals (Littleton, CO, USA).

### 2.2. Antibodies

The following primary antibodies were used: mouse anti-ß-Actin and mouse anti-FLAG (Sigma-Aldrich, St. Louis, MO, USA); mouse anti-Alix, mouse anti-EAP20, rabbit anti-EAP30, rabbit anti-EAP45, and mouse anti-Tsg101 (Santa Cruz Biotechnology, Dallas, TX, USA); mouse anti-GFP (BD Biosciences, Heidelberg, Germany); mouse anti-HA (Covance, Princeton, NJ, USA); and rabbit anti-Nedd4 (Cell Signaling, Danvers, MA, USA). Peroxidase-labeled, secondary antibodies were obtained from Dianova (Hamburg, Germany), and fluorophore-labeled antibodies were purchased from Molecular Probes (Eugene, OR, USA).

### 2.3. Cells and Transfection

For transient expression studies, the human hepatocellular carcinoma cell line HuH-7 (obtained from the European Collection of Authenticated Cell Cultures) and the human embryonic kidney 293T cell line (obtained from Luise Florin) were used. To transfect cells with siRNAs, the Lipofectamine™ RNAiMAX transfection reagent (Invitrogen, Carlsbad, CA, USA) was utilized. Briefly, 5 × 10^5^ cells per well of a 6-well plate were transfected with a final concentration of 30 nM siRNA according to the protocol of the supplier. After 48 h, cells were retransfected with 4 μg plasmid DNA using Lipofectamine™ Plus (Invitrogen), and transfected cells were harvested after additional 24 h. siRNA oligo sequences are provided in Supplemental Table S1.

### 2.4. Cell Lysis and Viral Particle Analysis

To probe for protein expression, cells were lysed with 1× Laemmli buffer, and cell suspensions were boiled for 10 min prior to centrifugation. Cell extracts were subjected to SDS-PAGE and Western blotting (WB) analyses using standard procedures. To analyze the assembly and release of viral particles from transfected cells, clarified culture medium was concentrated by ultracentrifugation through a 20% (*w*/*v*) sucrose cushion (4 h at 100,000× *g* and 4 °C). Pellets were suspended in 1× Laemmli buffer.

### 2.5. Quantitative Reverse Transcriptase (qRT)-PCR Analysis

Total mRNAs were isolated from cells using TRIzol Reagent (Life Technologies, Carlsbad, CA, USA) and Direct-zol MiniPrep (Zymo Research, Irvine, CA, USA), according to the protocols of the suppliers. The mRNA was treated with 5 U RNase-free, recombinant DNase I (Roche Diagnostics, Basel, Switzerland), and cDNA synthesis was performed by using the Transcriptor Universal cDNA Master Kit (Roche Diagnostics, Basel, Switzerland). For qRT-PCR, each reaction mixture (20 μL) contained 5 μL cDNA template, 1 μL forward primer (10 μM), 1 μL reverse primer (10 μM), 10 μL Fast Start Universal SYBR Green Probe Master (Roche Diagnostics), and 3 μL aqua bidest. Specific primer pairs were retrieved from PrimerBank [33], and sequences are available on request. PCR analyses were performed with a 7300 Real-Time PCR System and Sequence Detection Software v4.0 (Applied Biosystems, Waltham, MA, USA). For data analysis, the comparative cycle threshold method (∆∆Ct method) was used.

### 2.6. MLV Infection Assay

For the infection assay, HuH-7 cells were transfected with siRNAs for 48 h and retransfected with MLV.WT^FLAG/HA^ plus pCL-MFG-LacZ plasmid DNAs at a 1:1 DNA ratio. After 24 h, retroviral cell supernatants were harvested, filtrated (Ø 0.45 μm), and used immediately for infection. 24 h before infection, murine NIH 3T3 target cells were plated on cover slips at a density of 0.4 × 10^4^ cells per well of a 12-well plate and cultivated in DMEM supplemented with 10% fetal calf serum and 5 μg/mL Ciprofloxacin. The HuH-7–derived supernatants were titrated onto NIH 3T3 cells in the presence of 8 μg/mL polybrene. Two days post-infection, MLV infectivity was assayed by staining of cells for ß-galactosidase activity using the ß-Gal Staining Kit (Life Technologies) according to the supplied protocol. Cells were examined under a microscope by counting the number of blue cells and the total number of cells in eight random fields of view.

### 2.7. Fluorescence Microscopy

For immunostaining, siRNA- and DNA-transfected HuH-7 cells were treated with 1 μg/mL cycloheximide for 6 h to inhibit protein synthesis. Thereafter, cells were fixed and permeabilized with ice-cold methanol containing 2 mM EGTA. Cells were blocked in PBS containing 1% bovine serum albumin, incubated with the primary antibodies for 1 h at 37 °C, rinsed with PBS, and then incubated with AlexaFluor-conjugated secondary antibodies for 1 h at 37 °C. DNA was stained with Hoechst 33342 (Sigma-Aldrich). Images were acquired using a Zeiss Axiovert 200 M microscope equipped with a Plan-Apochromat 100× (1.4 NA, Zeiss, Oberkochen, Germany) and a Zeiss Axiocam digital camera (Zeiss). Axiovision software v4.7.1 (Zeiss) was used for merging pictures.

## 3. Results

### 3.1. Role of MVB Adaptors and ESCRT-I in MLV VLP and Virion Release

**Figure 1:**
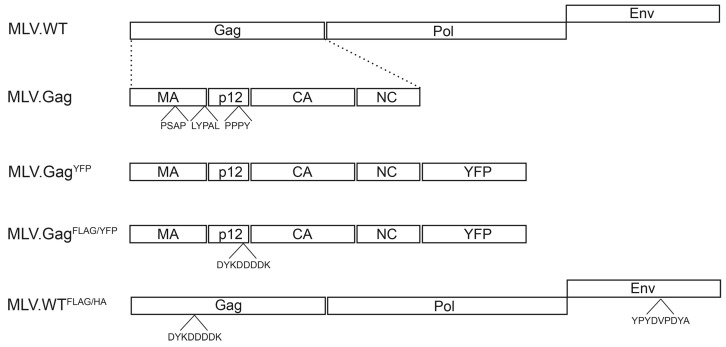
Schematic depiction of the MLV constructs. MLV.WT indicates the retroviral genome organization with the Gag, Pol, and Env ORFs of MLV. Below, the domain architecture of Gag with the matrix (MA), p12, capsid (CA), and nucleocapsid (NC) domains is depicted, and putative late domains (PSAP, LYPAL, and PPPY) are indicated. MLV.Gag^YFP^ carries the Gag ORF with a *C*-terminal YFP fusion, while MLV.Gag^FLAG/HA^ encodes an additional FLAG tag (DYKDDDK) within the p12 region. MLV.WT^FLAG/HA^ depicts a modified MLV genome carrying the FLAG tag within Gag and a HA epitope (YPYDVPDYA) within the ENV ORF.

The MLV Gag polyprotein contains three putative late domains located in the matrix (MA) domain, at the junction between MA and the p12 region, and in p12 [10,11,26] (Figure 1). Previous studies had shown that these late motifs mediate interaction with Tsg101, Alix, and HECT ubiquitin ligases, like Nedd4-1, Nedd4-2, WWP1, WWP2, and Itch [19,20,21,34]. To ease detection of synthesis and extracellular release of virus and virus-like MLV particles, human HuH-7 liver cells and human embryonic kidney 293T cells were used, that are not susceptible for specific binding and uptake of progeny MLV particles [26]. For transient expression in HuH-7 cells, a vector encoding the MLV Gag full-length precursor protein with a *C*-terminally fused YFP was used (Figure 1).

**Figure 2:**
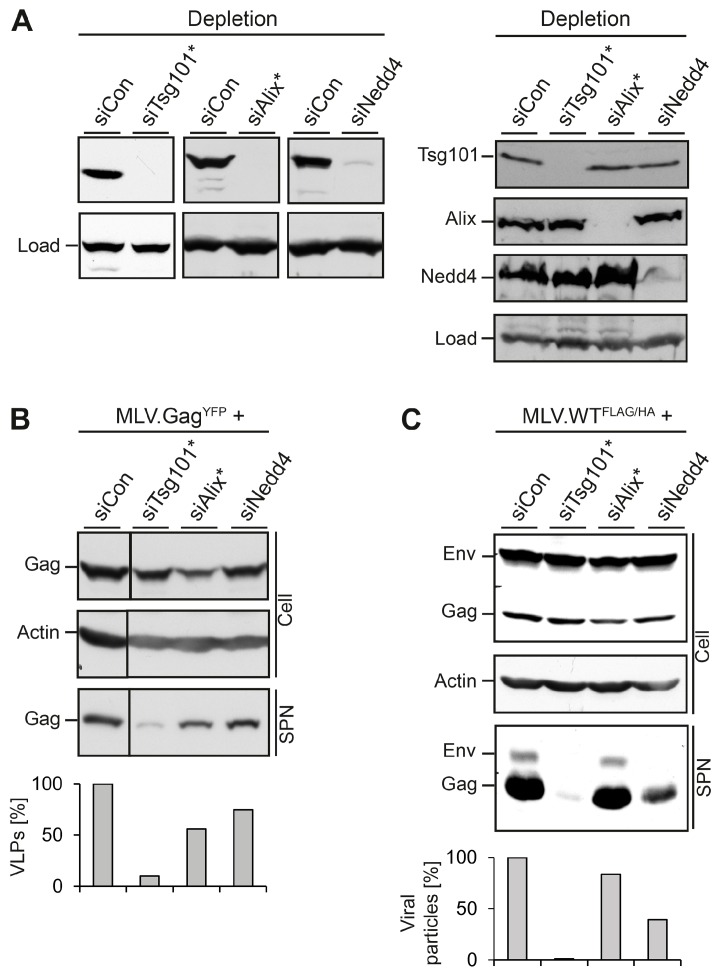
Impact of MVB adaptors and ESCRT-I on MLV VLP and virion release. (**A**) HuH-7 cells were treated with siRNA duplexes targeting Tsg101, Alix, Nedd4-1, or control siRNA for 48 h and were retransfected with the MLV constructs. In cases where single siRNA duplexes were used, the target gene is designated with a star. After transient expression for 24 h, lysates were subjected to Tsg101-, Alix-, and Nedd4-specific WB to monitor the efficiency of depletion/cross-depletion. Non-specific bands stained by the antisera served as controls for gel loading (Load); (**B**) The siRNA-treated cells were transfected with MLV.Gag^YFP^. Lysates (Cell) and VLPs released into the supernatants (SPN) were analyzed by anti-GFP WB. Uniformity of sample loading was probed by anti-ß-Actin WB. VLPs values were determined by densitometry and demonstrated in percent amount relative to control cells; (**C**) siRNA-treated cells were retransfected with the retroviral MLV.WT^FLAG/HA^ construct encoding FLAG-tagged Gag and HA-tagged Env proteins. Cell extracts and supernatants were analyzed by FLAG- and HA-specific WB. For a loading control, lysates were reacted with anti-ß-Actin antibodies. For quantification of extracellular virions, Env- and Gag-specific signals were measured by densitometry, summed, and demonstrated in percent amount relative to control cells.

First, we assessed whether the HuH-7 cell line confers the release of MLV.Gag^YFP^ in an ESCRT/MVB-dependent manner. Cells were treated with control siRNA or specific siRNAs directed against Tsg101, Alix, or Nedd4-1, prior to transfection with MLV.Gag^YFP^. For Tsg101, a single siRNA duplex was used, while Alix and Nedd4-1 were silenced with siRNA pools consisting of four different duplexes. Cell lysates were analyzed for the depletion efficacy of Tsg101, Alix, or Nedd4-1 by specific immunoblotting. All siRNAs effectively reduced the expression of their targets as compared to control siRNA-treated cells (Figure 2A, left panel). Since the knockdown of ESCRT or ESCRT-associated proteins eventually might trigger the breakdown of complex partner proteins, reminiscent of a so-called “co-depletion” phenomenon [17], we assessed the siRNA-treated cells for cross-depletion. Although Tsg101 and Alix are known interaction partners [1], depletion of Tsg101 did not reduce the level of Alix and *vice versa* (Figure 2A, right panel). Similar results were observed in siNedd4-1-treated cells, as the knockdown of Nedd4-1 neither affected Tsg101 nor Alix. To probe for the synthesis and release of MLV.Gag^YFP^, GFP-specific Western blotting (WB) was used. Except for cells in which Alix was knocked down, the intracellular level of MLV.Gag^YFP^ was largely unaffected (Figure 2B). When culture media of the cells were analyzed for VLP release, the individual loss of Tsg101 potently inhibited MLV.Gag^YFP^ egress, while the sole knockdown of Alix or Nedd4-1 reduced MLV.Gag^YFP^ export by about 44% and 25%, respectively (Figure 2B).

In parallel, we analyzed the roles of Tsg101, Alix and Nedd4-1 in the formation and egress of MLV viral particles. To this aim, we used an M-MLV retroviral vector encoding the Gag/Pol and Env ORFs of MLV. To facilitate detection of the viral proteins, the Gag polyprotein was tagged with a FLAG epitope, and a HA-tag was inserted into the Env ORF (see Figure 1). HuH-7 cells were transfected with control siRNA or siRNAs directed against Tsg101, Alix, or Nedd4-1, as above, and retransfected with the double-tagged MLV.WT^FLAG/HA^ construct. Cell lysates and concentrated supernatants were probed by FLAG- and HA-specific immunoblotting. The WB analysis of lysates showed that the depletions did not affect the synthesis and/or stability of the Env and Gag proteins as compared to siCon-treated cells (Figure 2C). Consistent with the results obtained for MLV VLPs, the depletion of Tsg101 very significantly reduced the release of virion-associated Env and Gag proteins. A similar inhibitory effect on MLV virion release was associated with the depletion of Nedd4-1, while the knockdown of Alix had only a minor effect on MLV.WT^FLAG/HA^ egress (Figure 2C). Notably, the effects of Alix and Nedd4-1 depletions on reductions in MLV virus-like and virus particle release were oppositional (Figure 2B,C), implicating the MLV particle types might differ in their budding requirements.

### 3.2. Role of ESCRT-II in MLV VLP and Virion Release

**Figure 3:**
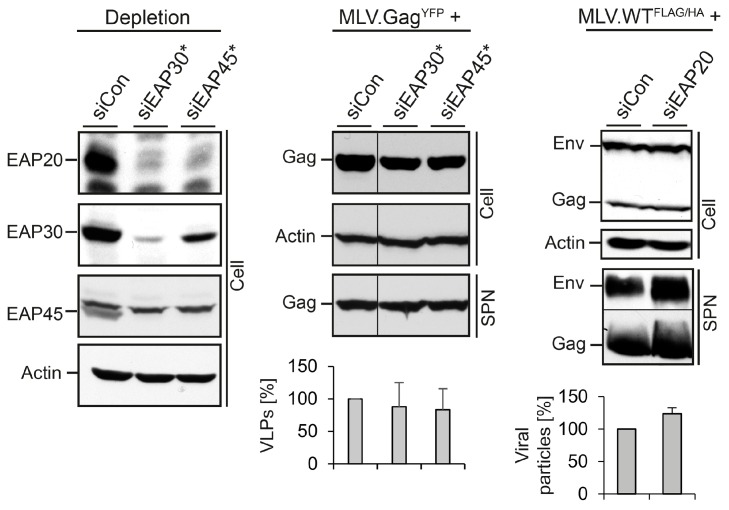
Impact of ESCRT-II on MLV VLP and virion release. Control siRNA or single siRNAs against EAP30 or EAP45 were introduced into HuH-7 cells prior to transfection with MLV.Gag. To assess depletion and co-depletion effects of the siRNAs, lysates were reacted with antibodies recognizing EAP20, EAP30, and EAP45. ß-Actin-specific WB was used to control equal gel loading (**left** panel). Cell-associated Gag and extracellular VLPs (SPN) were probed with anti-GFP WB (**middle** panel). VLPs values were determined by densitometry and demonstrated in percent amount relative to control cells (*n = 2*). Cells were treated with control siRNA or an EAP20-specific siRNA pool prior to transfection with MLV.WT^FLAG/HA^. Cell lysates and supernatants (SPN) were analyzed by FLAG/HA-specific WB and densitometric analyses (*n = 2*) (**right** panel) exactly as outlined in Figure 2C.

Next, we analyzed the role of the heterotetrameric ESCRT-II complex in MLV subviral and viral particle egress. ESCRT-II contains single copies of EAP45 and EAP30 as well as two copies of EAP20 and can bridge the ESCRT-I with the ESCRT-III complexes via interactions with the ESCRT-I subunit VPS28 and the ESCRT-III component CHMP6 [1,6,9]. Single siRNAs were used to silence EAP30 and EAP45 in cells expressing MLV.Gag^YFP^. Recently, we observed that these siRNAs not only reduced the expression of their targets, but also co-depleted the ESCRT-II complex partner proteins in HuH-7 cells replicating HBV [17]. To verify whether a similar phenomenon occurred upon MLV propagation, lysates were investigated using EAP20-, EAP30-, and EAP45-specific WB. As shown in Figure 3, the siRNAs against EAP30 and EAP45 down-regulated their targets and simultaneously provoked a cross-depletion of their complex partners, indicating that the knockdown of individual subunits of ESCRT-II triggers the breakdown of the entire complex. The production of MLV.Gag^YFP^ particles in ESCRT-II-depleted cells was monitored by GFP-specific WB. Thereby, we observed that the perturbation of ESCRT-II functions had no inhibitory effects on the formation and release of MLV.Gag^YFP^ VLPs (Figure 3, middle panel). Similar results were obtained when ESCRT-II-depleted cells were transfected with MLV.WT^FLAG/HA^. Here, an EAP20-specific siRNA pool was used to silence ESCRT-II. As shown by FLAG- and HA-specific WB of lysates and supernatants, EAP20 depletion did not affect the formation and release of MLV viral particles (Figure 3, right panel). Together, these data indicated that MLV does not depend on ESCRT-II function(s).

### 3.3. Role of ESCRT-III in MLV VLP Release

**Figure 4:**
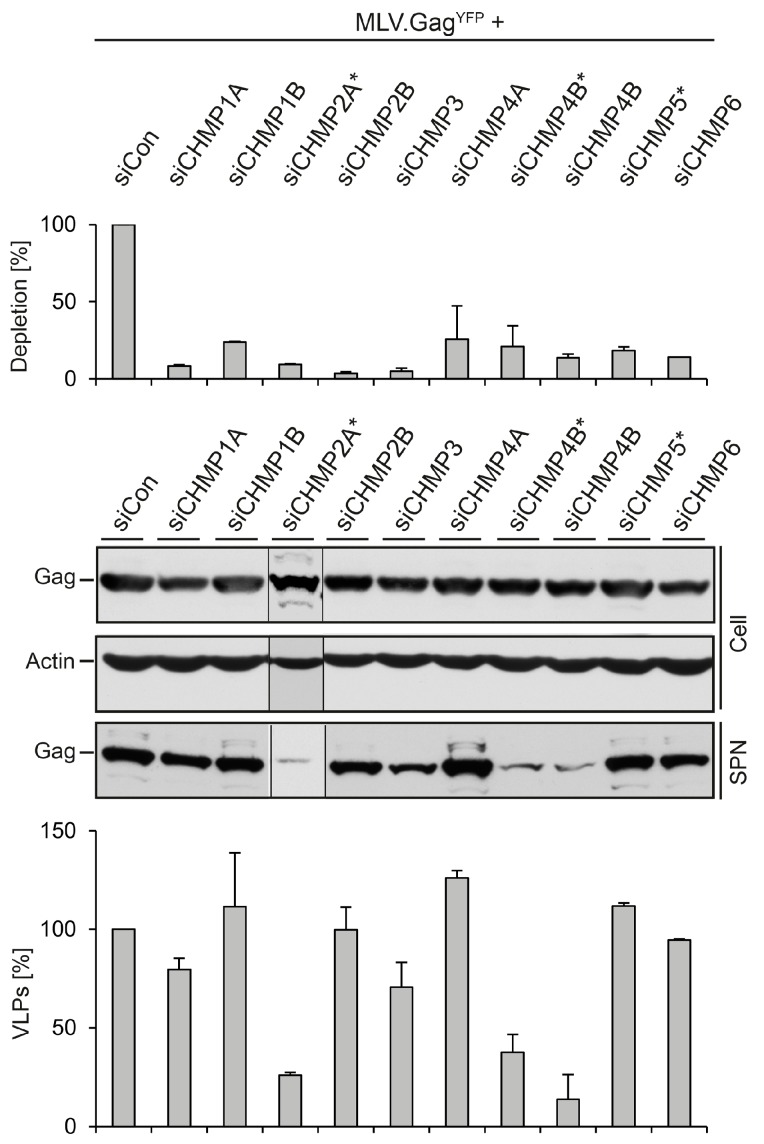
Impact of ESCRT-III family members on MLV.Gag^YFP^ release. HuH-7 cells were transfected with siRNAs targeting the indicated ESCRT-III genes and retransfected with MLV.Gag^YFP^. In cases where single siRNA duplexes were used, the target gene is designated with a star. The degrees of ESCRT-III knockdowns were measured by qRT-PCR. PCR runs were performed in duplicate, and error bars are standard error of the mean (**top** panel). Synthesis (Cell) and release (SPN) of MLV.Gag^YFP^ were probed by GFP-specific WB. Uniformity of sample loading was assessed by anti-ß-Actin WB (**middle** panel). VLPs values were quantified by densitometric analysis and demonstrated in percent amount relative to control cells in the graph below. Error bars indicate the standard deviations from the mean of two experiments (**bottom** panel).

Mammalian cells express 12 ESCRT-III-like proteins, designated CHMP1-7, and IST1, with three isoforms of CHMP4 and two isoforms each of CHMP1 and CHMP2. CHMP6, CHMP4 (A, B or C), CHMP3, and CHMP2 (A or B) are recruited in this order on membranes and are thought to polymerize into a helical architecture to constrict and abscise membrane buds. The remaining ESCRT-III (CHMP1A, B, CHMP5, CHMP7 and IST1) exerts regulatory functions, like mediating recruitment of the Vps4 ATPase. All ESCRT-III proteins are required for MVB biogenesis and during cell division [1,4,6,7,35]. Functional studies utilizing the overexpression of dominant-negative forms of some ESCRT-III subunits had shown that they globally inhibit ESCRT-dependent virus budding by entrapping the viral structural proteins in detergent-insoluble aggregates [3,5,10,36,37]. To determine the requirements of individual CHMP proteins for MLV virus-like and viral particle budding, we performed a systematic RNAi depletion analysis approaching most proteins except for CHMP4C, CHMP7, and Ist1. HuH-7 cells were treated with siRNA pools composed of four different duplexes directed against CHMP1A, CHMP1B, CHMP2A, CHMP2B, CHMP3, CHMP4A, CHMP4B, or CHMP6, followed by transfection with the MLV.Gag^YFP^ construct. For CHMP4B, a single siRNA duplex unrelated to the siRNA pool was applied in addition, while CHMP5 was targeted with a single siRNA duplex. Because commercial available antibodies against ESCRT-III components proved to be less suitable to clearly distinguish between the CHMP family members by immunoblotting, the silencing effects of the siRNAs were analyzed by qRT-PCR of reverse transcribed total mRNAs. Each siRNA compound proved to be effective and produced silencing effects of at least 70% as compared to siControl-treated cells (Figure 4). To investigate the fate of MLV.Gag^YFP^ in ESCRT-III-depleted cells, lysates and supernatants were probed using GFP-specific WB. As shown in Figure 4, neither knockdown grossly affected the intracellular level of MLV.Gag^YFP^. The quantification of VLPs released from the cells demonstrated that MLV.Gag^YFP^ clearly required the function of CHMP2A that could not be substituted by the CHMP2B isoform. In addition, the knockdown of CHMP4B, but not of CHMP4A, strongly diminished the extracellular level of MLV.Gag^YFP^. Moreover, VLP egress weakly involved the activity of CHMP3. Conversely, CHMP1A, CHMP1B, CHMP5, and CHMP6, as well as CHMP2B and CHMP4A, were found to be dispensable for VLP export (Figure 4). These results showed that, among the studied ESCRT-III proteins, only the inactivation of CHMP2A and CHMP4B led to a substantial decline of extracellular MLV.Gag^YFP^ particles.

### 3.4. Role of ESCRT-III in MLV Virion Release

**Figure 5:**
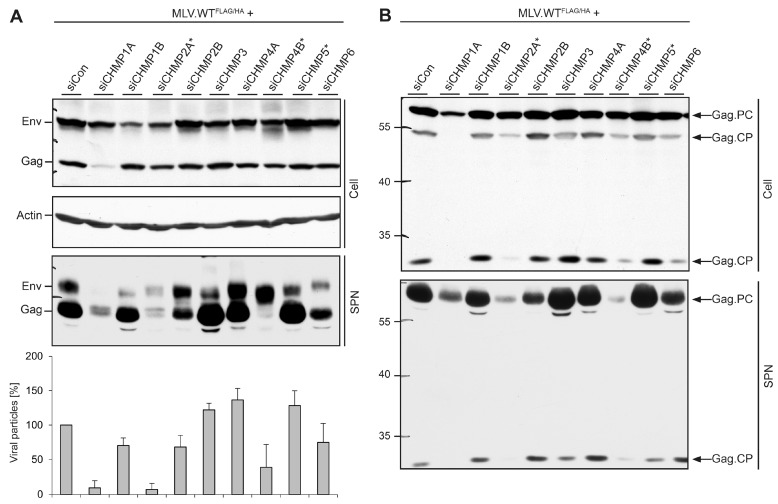
Impact of ESCRT-III family members on MLV virion formation, maturation, and release. (**A**) HuH-7 cells were treated with siRNAs targeting the indicated ESCRT-III genes, exactly as in Figure 4. Cells were retransfected with MLV.WT^FLAG/HA^, and lysates (Cell) and supernatants (SPN) were subjected to ß-Actin-, FLAG- and HA-specific WB and densitometric analyses (*n = 2*) as outlined in Figure 2C; (**B**) Lysates (Cell) and supernatants (SPN) derived from the same transfection as in (**A**) were run on separate gels and probed by FLAG-specific WB. Experiments were repeated twice and representative blots are shown. Arrows to the right indicate the Gag precursor protein (Gag.PC) and Gag-specific cleavage products (Gag.CP), and numbers to the left refer to molecular weight standards in kDa.

By using a similar approach, we studied the role of ESCRT-III in MLV virion production. In difference, siRNA-treated cells were transfected with the MLV.WT^FLAG/HA^ construct and cell extracts and supernatants were analyzed by tag-specific WB. The knockdowns of CHMP2B, CHMP3, CHMP4A, CHMP4B, CHMP5, and CHMP6 did not affect the overall degree of synthesis and stability of Env and Gag proteins within the cells as compared to siControl-treated cells (Figure 5A). By contrast, depletion of CHMP1A reduced the intracellular Gag level, while knockdowns of CHMP1B or CHMP2A slightly decreased the amounts of the Env proteins. Since the Gag/Pol and Env ORFs are all under the transcriptional control of the CMV immediate/early promoter in the MLV.WT^FLAG/HA^ construct, the observed reductions of Gag or Env in CHMP1A- or CHMP1B/2A-depleted cells, respectively, likely reflected disturbance in protein stability rather than differences in protein synthesis. Consistent with the results obtained for MLV.Gag^YFP^ (see Figure 4), the egress of viral particles strictly required the function of CHMP2A that could not compensated with CHMP2B (Figure 5A). Similarly, CHMP4B participated in MLV.WT^FLAG/HA^ release, whereas CHMP4A did not. Viral particles released from CHMP4B-depleted cells surprisingly contained higher amounts of Env and lesser amounts of Gag as compared to control cells, implicating that CHMP4B might play a particular role in Gag maturation (Figure 5A). The most striking phenotypes, however, were obtained for the CHMP1 proteins, as depletion of CHMP1A, but not of CHMP1B, almost completely impaired virion release. To verify the specific effect imposed by the lack of CHMP1A, it was important to rule out that the CHMP1A-specific siRNAs might co-deplete CHMP1B. However, as measured by qRT-PCR, CHMP1A-depleted cells contained 82.26% ± 3.46% (*n = 2*) CHMP1B-specific transcripts as compared to control cells.

To more closely explore the crucial roles of CHMP1A, CHMP2A, and CHMP4B in MLV virus production, we inspected the degree of Gag polyprotein processing. Lysates and supernatants of ESCRT-III-depleted, MLV-producing cells (see above) were solely analyzed by FLAG-specific WB. In control cells, Gag predominantly appeared as a 65 kDa species, corresponding to the full-length, uncleaved polyprotein, together with two species of 55 and 25 kDa (Figure 5B). Upon maturation, the MLV Gag precursor is known to be cleaved into MA, p12, capsid (CA), and nucleocapsid (NC) by the viral protease [26]. Considering the FLAG-labeling of the p12 domain of Gag, the 25 kDa-species likely represented a MA-p12 processing intermediate, while the 55 kDa-form might correspond to MA-p12-CA or p12-CA-NC cleavage products (Figure 5B). A comparable Gag processing pattern appeared in cells depleted for CHMP1B, CHMP2B, CHMP3, CHMP4A, and CHMP5 that were dispensable for MLV budding (Figure 5B). The depletion of the essential CHMP2A and CHMP4B subunits as well as the supportive CHMP6 protein led to a reduction of the Gag processing intermediates. Upon depletion of CHMP1A, an almost complete lack of proteolytic cleavage of Gag was evident (Figure 5B). The analysis of supernatants revealed a similar pattern for the 25 kDa-specific cleavage product. Even though the trace amount of the 65 kDa Gag precursor protein released from CHMP1A-depleted cells was higher as compared to that of CHMP4B-knockdown cells, no cleavage product was detectable in CHMP1A-depleted cells (Figure 5B). Hence, CHMP1A might play an additional, non-canonical ESCRT-III function during MLV virion maturation.

### 3.5. Divergent Role of CHMP1A in MLV VLP and Virion Release

**Figure 6:**
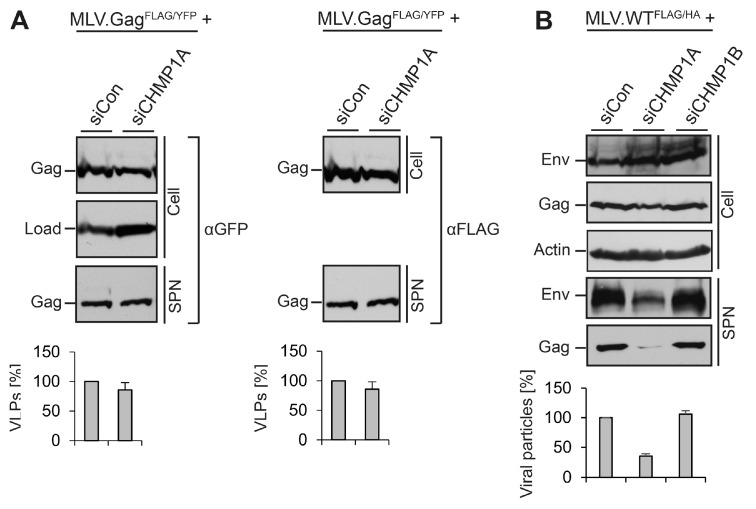
Validation of the role of CHMP1A in viral, but not virus-like MLV particle release. (**A**) CHMP1A is dispensable for the release of FLAG-tagged MLV.Gag^FLAG/YFP^. HuH-7 cells were treated with control siRNA or siRNA pools targeting CHMP1A. Cells were transfected with an YFP-tagged MLV.Gag construct carrying a FLAG tag within the p12 domain. MLV.Gag^FLAG/YFP^ expression (Cell) and release (SPN) were assayed by FLAG-specific WB prior to GFP-specific immunoblotting of the same blot. Non-specific bands stained by the antisera served as controls for gel loading (Load). The degree of VLP release was quantified by densitometric analysis and demonstrated in percent amount relative to control cells (*n = 2*); (**B**) CHMP1A is essential for MLV virion release in HEK 293T cells. Following treatment of HEK 293T cells with control siRNA or siRNA pools against CHMP1A or CHMP1B, they were transfected with MLV.WT^FLAG/HA^. Intra- and extracellular Env and Gag levels were determined by anti-HA and anti-FLAG WB on separate blots. The anti-ß-Actin blot served as a loading control. For quantification of extracellular virions, Env- and Gag-specific signals were measured by densitometry, summed, and demonstrated in percent amount relative to control cells (*n = 2*).

To gain insights into the pivotal role of CHMP1A in MLV virus *versus* VLP morphogenesis, it was also important to exclude the possibility that the FLAG-tag introduced into the p12 domain of Gag somehow might contribute to the CHMP1A dependency. Therefore, we constructed an YFP-tagged MLV.Gag construct carrying an analogous FLAG epitope insertion (MLV.Gag^FLAG/YFP^) and analyzed its behavior in siControl- and siCHMP1A-depleted cells. By probing cell lysates and supernatants by FLAG- and GFP-specific WB, we found that the CHMP1A knockdown did not impair the synthesis and release of double-tagged MLV VLPs (Figure 6A). We therefore ruled out that the FLAG insertion accounted for the CHMP1A requirement in MLV virus production.

For other retroviruses, like HIV-1, host cell requirements might vary depending on the cell type used for virus propagation [8,16]. To assess the CHMP1A dependency of MLV virions in another cell line, we conducted experiments in HEK 239T cells, human embryonic kidney cells. HEK 293T cells were transfected with Control-, CHMP1A-, or CHMP1B-specific siRNAs, retransfected with the MLV.WT^FLAG/HA^ vector, and analyzed as above. Consistent with the results obtained with HuH-7 cells, depletion of CHMP1A, but not CHMP1B, severely blocked virus release (Figure 6B).

**Figure 7:**
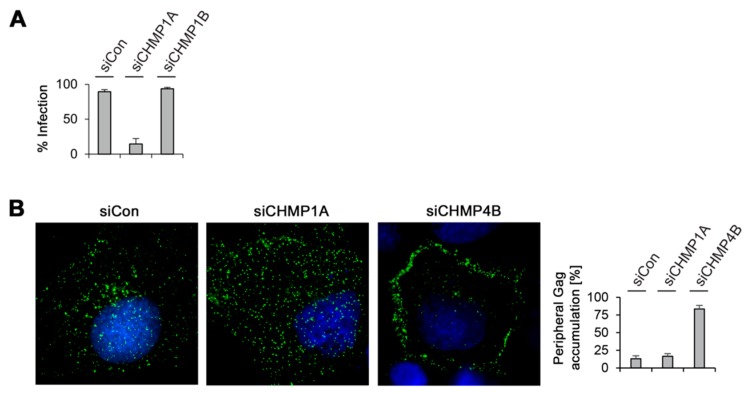
MLV infectivity and intracellular distribution in CHMP-knockdown cells. (**A**) Depletion of CHMP1A, but not of CHMP1B, decreased MLV infectivity. Control-, CHMP1A-, and CHMP1B-knockdown HuH-7 cells were cotransfected with MLV.WT^FLAG/HA^ plus a pCL-MFG-LacZ reporter plasmid. Viral particles released into the supernatants were harvested 24 h after DNA transfection and transduced into murine NIH 3T3 cells. Cells were stained for ß-galactosidase activity two days later, quantitated, and averaged from two independent experiments; (**B**) Control-, CHMP1A-, and CHMP4B-knockdown HuH-7 cells were transfected with MLV.WT^FLAG/HA^ and subjected to immunostaining with mouse anti-FLAG antibodies. Following staining with AlexaFluor 488-conjugated anti-mouse antibodies, cells were analyzed by fluorescence microscopy and representative images are shown. DNA staining is shown in blue. For quantification, the Gag distribution pattern was inspected in about 50 cells from two independent experiments and plotted in the graph.

To obtain further clues of the different roles of CHMP1A and CHMP1B in MLV virus propagation, we performed infectivity studies. Previous studies demonstrated that maturation inside the immature MLV virion is required for infectivity [26,38,39]. Accordingly, the yield of extracellular virions might not be a mandatory indicator of virus infectivity. To ascertain the seemingly dispensable role of CHMP1B, we measured the infectivity of virions released from CHMP1B-depleted HuH-7 cells. Cells were treated with Control- and CHMP1B-specific siRNAs, as well as with siRNAs against CHMP1A, and retransfected with the MLV.WT^FLAG/HA^ vector together with a ß-galactosidase reporter plasmid. Viral particles harvested from the supernatants were then used to infect NIH 3T3 target cells and MLV infectivity was quantitated by staining cells for ß-galactosidase activity. As would be expected from the very low virus yield from CHMP1A-depleted cells, the titer of infectious particles was correspondingly depressed (Figure 7A). Importantly, the down-regulation of CHMP1B did not reduce MLV.WT^FLAG/HA^ infectivity as compared to viral particles produced in control cells (Figure 7A). These data indicate that CHMP1B is not essential for MLV virus release and infectivity, whereas its closely related CHMP1A paralog is.

Finally, immunofluorescence microscopy studies were performed in order to study the intracellular Gag distribution when virus export is blocked by CHMP1A or CHMP4B inactivation. HuH-7 cells were treated with control siRNA or duplexes against CHMP1A or CHMP4B and transfected with the MLV.WT^FLAG/HA^ construct. For protein synchronization, cells were treated with cycloheximide before fixation. The CHMP4B-specific knockdown was included as a reference, as it blocked both, MLV VLP and viral particle release. Cells were stained with FLAG-specific antibodies to visualize the distribution of Gag. In control cells, Gag yielded a punctuate staining pattern distributed throughout the cytoplasm (Figure 7B). Upon CHMP4B depletion, the distribution of Gag changed, as it accumulated in the cell periphery, reminiscent for the plasma membrane. In contrast, such a peripheral accumulation of Gag was not detectable in CHMP1A-depleted cells (Figure 7B), implicating that Gag might fail to be transported to the budding site.

## 4. Discussion

Several studies had shown that MLV budding depends on a functional ESCRT machinery, but the exact roles of different ESCRT factors remain unknown [19,20,21,34]. Our functional analyses reveal that MLV subviral and viral particle release requires only a small subset of ESCRT subunits, namely the ESCRT-I subunit Tsg101 and the ESCRT-III subunits CHMP2A and CHMP4B along with supportive roles of the ESCRT/MVB-associated factors Alix and Nedd4-1. The budding pathways of MLV virions and VLPs share the selective utilization of these components, but differ in their unequal use of the ESCRT-III factor CHMP1A. This finding was surprising, because in general full particles and VLPs of a given retrovirus are thought to engage similar ESCRT factors for budding. However, as exemplified by HBV, an enveloped pararetrovirus, and Sendai virus, a member of the paramyxovirus family, the budding pathways of infectious virions and subviral particles differ in their ESCRT requirements [40,41,42]. Therefore, we reasoned to comparatively study host cell assistance in MLV virion and VLP release.

We observed, as have others, that the MLV Gag polyprotein gain access to the ESCRT machinery by means of its three late domains and their respective binding partners [19,20,21,34]. Among the late domains, the PPPY motif in the MLV Gag protein had been shown to be essential for virus egress, while the PSAP and LYPAL motifs exert complementary budding activities [19,25]. Given the importance of the PPPY domain, the moderate MLV budding defect observed in Nedd4-1-depleted cells was surprising, but likely due to the redundant action of Nedd4 family proteins and associated ART adaptor proteins in MLV release [20,21,34]. Consistent with a previous work [19], the most potent block in MLV egress occurred in Tsg101-knockdown cells, despite the ancillary role of the PSAP motif in virus release. The critical role of Tsg101 may be interconnected with the PPPY domain of MLV, as Nedd4/HECT ubiquitin ligases had been shown to bind and ubiquitinate ARTs which in turn can interact with the ubiquitin binding domain of Tsg101, thereby activating ESCRT-I to function in virus budding [21,24].

Unexpectedly, we found that MLV budding does not need ESCRT-II. Viruses that encode a single PPXY motif, such as ASV and HBV, had been shown to require ESCRT-II functions for maturation and release [17,18,43], suggesting that sole viral PPXY motifs may direct the preferential use of ESCRT-II. Possibly, such an ESCRT-II dependency may be abrogated, if the PPXY motif is combined with additional late domains, as is the case in MLV. The ESCRT-II independency of MLV matches our observations that budding of VLPs and virions did not or only marginally, respectively, depend on CHMP6. CHMP6 is the only ESCRT-III factor known to bind ESCRT-II, thereby initiating the polymerization of ESCRT-III subunits on endosomal membranes during MVB biogenesis. This poses the question, how MLV triggers ESCRT-III assembly via Tsg101/ESCRT-I. A similar phenomenon had been observed in the course of budding of HIV-1 that enters the ESCRT pathway via a P(T/S)AP/Tsg101-interaction and activates ESCRT-III without an apparent need of ESCRT-II and CHMP6 [8,44]. This had been interpreted in such that HIV-1 Gag and/or ESCRT-I appear to recruit CHMP4 using additional protein-protein or protein-membrane interactions that remain to be identified [44].

Beside the ESCRT-III factor CHMP6, we found that CHMP1B and CHMP5 family members, known to be involved in MVB sorting and cytokinesis [1,6,45], also do not assist in MLV egress. Rather, among the ESCRT-III factors tested, only CHMP2A and CHMP4B turned out to be essential for both, MLV virion and VLP budding, while CHMP3 may play a slight supporting role in VLP release. Interestingly, CHMP2B and CHMP4A failed to compensate for the loss of their counterparts. In case of the CHMP4 isoforms, the functional superiority of CHMP4B may parallel its reported cellular abundance and/or its preferred binding by Alix [46], which, among others, is utilized by MLV.Gag to enter the ESCRT machinery. For CHMP2 family members, structural and functional differences have been reported, as e.g., CHMP3 synergizes more efficiently with CHMP2A than with CHMP2B [47,48]. Among others, this may account for the preferential use of the CHMP2A isoform by MLV. The essential function of CHMP2A is likely attributed to its capacity to recruit the VPS4 ATPase that provides the driving force for ESCRT-III disassembly and for multiple cycles of virus release [1,7]. For the recruitment of VPS4, ESCRT-III subunits interact with the microtubule-interacting and transport (MIT) domain of the enzyme via MIT-interacting motifs (MIMs) [49]. Among the CHMP proteins, the yeast CHMP2 ortholog, *Vps2*, had been shown to have the highest affinity for VPS4 [50]. Another clue for the important role of CHMP2A in MLV egress may be its ability to interact with LIP5, a positive regulator of VPS4, that promotes VPS4 oligomerization and activation [51]. LIP5 also binds CHMP5, but this interaction strongly inhibits LIP5-mediated VPS4 stimulation in mammalian cells [52]. This may account somehow for our finding that CHMP5 is not required for MLV budding.

Our data are consistent with two reports in which the ESCRT requirements for HIV-1 and EIAV viral particle budding and viral infectivity were studied [44,53]. Like MLV, both viruses strictly require one CHMP2 and one CHMP4 family member with preferences for CHMP2A and CHMP4B. To assist in HIV-1 and EIAV budding, an interaction between CHMP2 and CHMP4 isoforms is mandatory. This led to the proposal that CHMP4 assembles first within the neck of the budding virion, where it can recruit CHMP2 by direct interaction followed by VPS4 recruitment leading to virion fission [44,53]. In further agreement with our data, CHMP5 and CHMP6 are also dispensable for HIV-1 production [8,44,45]. Despite their similar budding requirements, HIV-1, EIAV, and MLV differ in subtle but peculiar demands. While EIAV release does not depend on CHMP3 [53], this ESCRT-III factor plays an auxiliary role in MLV VLP (this work) and HIV-1 egress [44]. Furthermore, CHMP1 family members are engaged differently, as HIV-1 requires the specific function of CHMP1B [44], while MLV virus production needs CHMP1A, as shown herein.

Interestingly, a non-canonical ESCRT pathway has been recently described that is used for down-regulation of virally ubiquitinated major histocompatibility complex (MHC) class I via the MVB. Unlike conventional MVB sorting, this pathway neither needs ESCRT-II nor CHMP6 but operates with the histidine domain phosphotyrosine phosphatase, a Bro1 domain-containing protein related to Alix. Similar to MLV budding, MHC class I sorting only requires a subset of ESCRT-III factors, like CHMP2A, CHMP4B, and CHMP3, whereas CHMP2B, CHMP4A, and CHMP4C are not involved [54]. Hence, it is tempting to speculate that virus budding pathways may reflect phenocopies of minimal cell fission events.

One unexpected finding of this work was the observation that CHMP1A is a dependency factor of MLV virion, but not VLP egress. In general, full particles and VLPs of one retrovirus are thought to exploit identical ESCRT factors for budding. To account for the differential functions of the two CHMP1 isoforms in MLV release, isoform-specific modifications may be involved, as CHMP1A had been shown to be a target for phosphorylation and sumoylation, and contains a putative ubiquitin-interacting motif [55,56,57]. Modification-dependent functions had been described for the CHMP4 isoforms, as only CHMP4C can be phosphorylated by the Auro B kinase in order to control timing of cell abscission during cytokinesis [58]. Although the role of CHMP1A in MLV budding remains to be completely defined, CHMP1A-knockdown cells displayed a defect/delay in Gag polyprotein processing and/or stability along with a diminished Gag accumulation in the cell periphery that is normally reminiscent for virions arrested in the final budding stage. Hence, it appears conceivable that CHMP1A may play an adaptor-like role in proper trafficking and targeting of Gag and Gag/Pol polyproteins to the MLV budding site where the viral protease comes into play. Understanding how CHMP1A mechanistically operates during MLV maturation will be an important next step forward.

## 5. Conclusions

In summary, our experiments reveal that MLV subviral and viral particle budding depends on a surprisingly small subset of ESCRT proteins, including TSG101, CHMP2A, and CHMP4B. MLV virions and VLPs share the minimal requirements for these ESCRT factors, but differ in their unequal use of CHMP1A. Albeit the fundamental role of CHMP1A played in MLV virus release remains to be elucidated, our results provide first evidence that CHMP1A may function in virus maturation and trafficking processes.

## Supplementary Materials

The following is available online at www.mdpi.com/1999-4915/8/4/103/s1, Table S1: siRNAs used in this study.

## Abbreviations

The following abbreviations are used in this manuscript:

MLV	Murine leukemia virus
ESCRT	endosomal sorting complex required for transport
VLP	viral-like particles
MVB	multivesicular body
HIV-1	human immunodeficiency virus type 1
RSV	Rous sarcoma virus
HBV	Hepatitis B virus
ASV	Avian sarcoma virus
EIAV	equine infectious anemia virus
ART	Arrestin-related trafficking
YFP	yellow fluorescent protein
Pol	Polymerase
Env	Envelope
ORFs	open reading frames
aa	Amino acid
WB	Western blotting
qRT	quantitative reverse transcriptase
MA	Matrix
CA	Capsid
NC	Nucleocapsid
MIT	Microtubule-interacting and transport
MIM	MIT-interacting motifs
