# Supplementary material for: ESCRT Requirements for Murine Leukemia Virus Release
Source: Viruses. 2016 Apr 18;8(4):103. doi: 10.3390/v8040103 (PMC4848597; doi:10.3390/v8040103)
Supplement: Supplementary file 1 [file viruses-08-00103-s001.pdf]

Supplementary Materials: ESCRT Requirements for Murine Leukemia Virus Release

Christina Bartusch and Reinhild Prange

Table S1. siRNAs used in this study.

Gene description	siRNA Target Sequence
Tsg101 [1]	CUCAAUGCCUUGAAACGAA
Alix [1]	GCAGUAAUAUGUCUGCUCA
Nedd4-1	GGGAUUCUUUGAACUAAUA
Nedd4-1	GGAGGGAACAUACAAAGUA
Nedd4-1	GAUCACAAUUCCAGAACGA
Nedd4-1	GAUCACAAUUCCAGAACGA
EAP20	GUCGAUCCAGAUUGUAUUA
EAP20	GGGAAACUCAUCUAUCAGU
EAP20	GCACAAGGCCGAGAUCAUC
EAP20	CAGAACAACUCCGUCUUUA
EAP30 [1]	GAAUGGAGGUCUGAUAACU
EAP45 [1]	GGAAUAUUGCAGGUGCCUU
CHMP1A	GAAGGUCUCCUCAGUGAUG
CHMP1A	GAGAACGCCAUCCGCAAGA
CHMP1A	CACCGCGUCUUGCCUUUGU
CHMP1A	GGUGUUGAGUUUCUGCAAA
CHMP1B	GAAGAUUUCUGCUUUGAUG
CHMP1B	GGAGCAGGAUGAACUGUCU
CHMP1B	GAUAUGCUGCUCCAGGAAA
CHMP1B	GAGGAUACACGCCGAAAAU
CHMP2A	AGGCAGAGAUCAUGGAUAU
CHMP2A	GCGCAAGUUUGUAUUGAUG
CHMP2A	UUAAGAAGAUGGCCAAGCA
CHMP2A	CAGAUGAGCUGUCGAACCU
CHMP2B	GAAGAUGGCUGGAGCAAUG
CHMP2B	UAAGGAAGCUUGCAAAGUU
CHMP2B	GCUCGAAGCUUACCAUCUG
CHMP2B	GCCAGGAUAUUGUGAAUCA
CHMP3	UGACAGAAUUCUCUUUGAA
CHMP3	GAAGAGCACAGAAGUGAUG
CHMP3	GGGCAAAGCACCCAGUAAA
CHMP3	AGAACCAGCUCGCGGUCUU
CHMP4A	CAAGGUAGAUGAACUGAUG
CHMP4A	UAAGAGAGCUGCCCUACAG
CHMP4A	GGAAUUGGCCCAGGAGUUG
CHMP4A	GAAGCAAUACAGAAACUGA
CHMP4B	GCAGAUCGACGGCACAUUA
CHMP4B	GGACAUCGAUAAAGUUGAU
CHMP4B	GGAAAUCAGUGGACCCGAA
CHMP4B	GGACACGGAAGAGAUGUUA
CHMP4B [1]	GGCUAUGCCGCCAAGGCCA
CHMP5 [1]	CAGAAAGCCUUGCGAGUUU
CHMP6	UCACCCAGAUCGAAAUGAA
CHMP6	CGCAAUCACUCAGGAACAA

CHMP6	GAGUACCAGCGGCAAAUAG
CHMP6	CGGAUUAACUCUCGACCGA

[1] siRNA was applied in single format.

© 2016 by the authors; licensee MDPI, Basel, Switzerland. This article is an open access article distributed under the terms and conditions of the Creative Commons by Attribution (CC-BY) license (http://creativecommons.org/licenses/by/4.0/).
